# Retraction: Design of 1-methylimidazolium tricyanomethanide as the first nanostructured molten salt and its catalytic application in the condensation reaction of various aromatic aldehydes, amides and β-naphthol compared with tin dioxide nanoparticles

**DOI:** 10.1039/d2ra90090d

**Published:** 2022-09-26

**Authors:** Mohammad Ali Zolfigol, Saeed Baghery, Ahmad Reza Moosavi-Zare, Seyed Mohammad Vahdat, Heshmatollah Alinezhad, Mohammad Norouzi

**Affiliations:** Faculty of Chemistry, Bu-Ali Sina University Hamedan 6517838683 Iran zolfi@basu.ac.ir mzolfigol@yahoo.com +98 8138257407; Department of Chemistry, University of Sayyed Jamaleddin Asadabadi Asadabad 6541835583 Iran moosavizare@yahoo.com; Department of Chemistry, Ayatollah Amoli Branch, Islamic Azad University P.O. Box 678 Amol Iran; Faculty of Chemistry, University of Mazandaran Babolsar 47415 Iran; Department of Chemistry, Payame Noor University P.O. Box 19395-3697 Tehran Iran

## Abstract

Retraction of ‘Design of 1-methylimidazolium tricyanomethanide as the first nanostructured molten salt and its catalytic application in the condensation reaction of various aromatic aldehydes, amides and β-naphthol compared with tin dioxide nanoparticles’ by Mohammad Ali Zolfigol *et al.*, *RSC Adv.*, 2015, **5**, 45027–45037, https://doi.org/10.1039/C5RA02718G.

The Royal Society of Chemistry hereby wholly retracts this *RSC Advances* article as the synthesis of 1-methylimidazolium tricyanomethanide reported in the article, whereby tricyanomethane is used as a starting material, is not reproducible. The authors stated that they did not report the synthesis of tricyanomethane in the published paper as they purchased this compound from a commercial center and used it in the synthesis of ionic liquids, molten salts and various kinds of catalysts. The authors thought the reaction between tricyanomethane and organic bases is a simple acid–base reaction, therefore they did not cite the previously reported literature and its related history for the preparation of tricyanomethane. However, according to papers by Banert *et al.*,^1,2^ and based on their obtained analysis of the chemical sold to them as tricyanomethane, it became clear to the authors that this purchased compound was not tricyanomethane as there were differences in the ^1^H NMR and ^13^C NMR chemical shift between the purchased compound and the reports of Banert *et al.*^1^ According to these documents, the authors believe that the compound sold to them as tricyanomethane was fake. While the authors have now re-prepared 1-methylimadazolium tricyanomethanide, by synthesising potassium tricyanomethanide as a starting material for the synthesis,^3–5^ the synthesis reported in this article is not accurate. Therefore, this article is being retracted to avoid misleading readers and to protect the accuracy and integrity of the scientific record.

Mohammad Ali Zolfigol, Saeed Baghery and Ahmad Reza Moosavi-Zare oppose the retraction. Seyed Mohammad Vahdat, Heshmatollah Alinezhad and Mohammad Norouzi were contacted but did not respond.

Signed: Laura Fisher, Executive Editor, *RSC Advances*

Date: 17^th^ August 2022

## Supplementary Material
